# Human attachment as a multi-dimensional control system: A computational implementation

**DOI:** 10.3389/fpsyg.2022.844012

**Published:** 2022-09-15

**Authors:** Marcantonio Gagliardi

**Affiliations:** Department of Computer Science, The University of Sheffield, Sheffield, United Kingdom

**Keywords:** attachment, mother-child, dimension, representation, control system, agent-based model, simulation, strange situation

## Abstract

Attachment is an emotional bond between two people where one seeks care from the other. In the prototypical case, the child attaches to their mother. The most recent theoretical developments point out that attachment is multidimensional – meaning that the phenomenon pertains to multiple domains related to the relationship with the caregiver. However, researchers have so far modeled attachment computationally by mostly adopting a classical categorical (as opposed to dimensional) standpoint that sees the system as controlling caregiver proximity. In contrast, we adopt here a *dimensional perspective* (DP) and consider dimensions to be the system’s set-goals. We hypothesize that the resulting multidimensional controller should lead to valid (or even better) models of the phenomenon. To start testing this hypothesis, we built a DP-informed agent-based model of attachment inspired by the widely-studied Strange Situation Procedure. In this context, child and mother show the nature of attachment bonds through their behavioral and emotional expressions. By modeling them as point-agents moving in a two-dimensional arena, we simulated child-mother interactions for the avoidant and ambivalent attachment dimensions. The generated dynamical patterns – characterized by the alternation between approach and exploration – matched those described in the attachment literature, thereby confirming the implementability and validity of the DP.

## Introduction

Attachment is a psychological phenomenon that manifests itself as a particular emotional bond between two humans where one of them, whom we will refer to as the *attacher*, attaches to the other, the *caregiver*, who provides care (Bowlby, 1969/1982). The topic is complex and covered by the vast corpus of literature known as attachment theory (Cassidy and Shaver, 1999, 2008, 2016). In this work, we address the problem of capturing and testing the dimensional nature of attachment through computational modeling.

So far, following a classical theoretical approach, computational models of attachment have focused on attachment behavior as driven by the set-goal of proximity (Bowlby, 1969/1982; Petters, 2019). In contrast, we present here the Dimensional Attachment Model (DAM), a computational model of attachment that represents the most recent *dimensional perspective* (DP) on attachment, according to which attachment is primarily about building a multi-dimensional relationship (Fraley and Spieker, 2003; Roisman et al., 2007; Liotti and Farina, 2011; Sherman et al., 2015; Gagliardi, 2021, 2022). By investigating it computationally, we demonstrate that this theoretical perspective (1) is implementable and (2) can lead to valid simulations of attachment phenomena (i.e., simulations compliant with the psychological data). We start by outlining the relevant theory, then describe our model, and, finally, discuss its contribution and limitations.

## Attachment theory

The attachment relationship is as central in human psychological life (Bowlby, 1969/1982, 1973, 1980) as it is difficult to conceptualize, as demonstrated by the core issues that are still controversial in attachment theory – for example, intergenerational transmission (Verhage et al., 2016; van Ijzendoorn and Bakermans-Kranenburg, 2019), stability (McConnell and Moss, 2011; Pinquart et al., 2013), relationship with psychopathology (DeKlyen and Greenberg, 2016; Stovall-McClough and Dozier, 2016). However, an advantageous conceptualization is essential to effective modeling. Therefore, we outline here the concepts most relevant to such an endeavor, considering that the two main characteristics of attachment are (1) the innate motivation to attach and (2) the information acquired in the process.

### Attachment as a motivational system

Broadly speaking, humans are driven by a set of intrinsic motivations that promote activities such as eating, regulating body temperature, mating, exploring, cooperating, etc., and attaching (seeking care) and caregiving belong to this set. It is widely supposed that each human motivation corresponds to a motivational system located in the brain (Bowlby, 1969/1982; Lichtenberg et al., 2010; Panksepp and Biven, 2012; Liotti et al., 2017; Schaller et al., 2017) and interacts with the others to generate the motivational dynamics that underpins human action. With regards to early attachment relationships, besides *attachment* and *caregiving*, *exploration* is the most relevant motivational system (Bowlby, 1969/1982; Ainsworth et al., 1978).

Although the prototypical attachment relationship is the one between child and mother, such a relationship can be formed between any two people over the entire course of life (Bowlby, 1969/1982; Marvin et al., 2016; Mikulincer and Shaver, 2016; Fraley and Roisman, 2019), literally *“from the cradle to the grave”* (Bowlby, 1969/1982, p. 208). In all cases, the attacher is intrinsically motivated to ask for care (i.e., attach), and the caregiver is intrinsically motivated to provide it (Bowlby, 1969/1982, 1973, 1980). At the beginning of their explorative development, the child appears to maintain a proper balance between attachment and exploration by keeping their caregiver as a secure base for exploration (Ainsworth et al., 1978; Waters and Waters, 2006). The model we present – the DAM – reproduces attachment interactions between a child, driven by attachment and exploration, and a caregiver (that can be thought of as a mother), driven by caregiving and exploration.

### Attachment information and its manifestation

Attachment is an adaptation mechanism essential to survival and reproduction, which relies on the acquisition of fundamental information (Bowlby, 1969/1982; Chisholm and Sieff, 2014; Simpson and Belsky, 2016; Szepsenwol and Simpson, 2019) – acquired representations of the other and the self usually called Internal Working Models (Sherman et al., 2015; Marvin et al., 2016). Since infancy, such information manifests itself in patterns of behavior and internal states that have been studied by adopting two different perspectives: categorical and dimensional.

#### Categorical perspective

Attachment was first identified in children and measured through an experimental paradigm known as the *Strange Situation Procedure* (SSP) (Ainsworth et al., 1978; Main and Solomon, 1990). The SSP is realized in a room where the child is the protagonist of eight three-minute episodes in which they are either alone or can interact with a caregiver or a stranger, allowing for the child’s attachment to be elicited. Through this procedure, four categories of attachment – *security*, *avoidance*, *ambivalence*, and *disorganization* – have been identified as corresponding to precise behavioral and emotional patterns expressed by the child during the eight episodes (Hesse, 2008). In this scheme, security simply means the absence of both avoidance and ambivalence, while disorganization is seen as a particular condition. Importantly, this conceptualization identifies a “security-insecurity dimension” and a corresponding caregiving feature – often called “sensitive responsiveness” – as underlying both avoidance and ambivalence (Ainsworth et al., 1978, p. 152). In other words, avoidance and ambivalence are seen as two opposite manifestations of the same dimension. The success of the SSP has been consolidated by the Adult Attachment Interview (AAI) (George et al., 1985; Main and Hesse, 1990; Hesse, 2016), through which the state of mind with respect to attachment can be measured in adults. The AAI identifies four attachment styles that correspond to the patterns identified by the SSP, thereby supporting the persistence of attachment phenomena throughout life. These kinds of measures consider attachment as characterized by mutually exclusive categories.

#### Dimensional perspective

Although the categorical view of attachment is still in use, further research has shown that attachment can be better characterized as a multidimensional phenomenon. In particular, the four identified categories can be described by *three (relatively) independent dimensions* that correspond to representations acquired on specific aspects of the relationship (Fraley and Spieker, 2003; Liotti, 2011; Fraley et al., 2015; Paetzold et al., 2015; Mikulincer and Shaver, 2016; Gagliardi, 2021). Following the SSP, we refer to these dimensions as *avoidance, ambivalence*,^1^ and *disorganization*. They can fully express the range of behaviors and internal states detectable through the SSP at around 1 year of age. Attachment disorganization has been connected to the experience of a frightening caregiver (Main and Solomon, 1990; Lyons-Ruth and Jacobvitz, 2016). In the SSP, disorganized children typically express incoherent/contradictory behaviors that arise from the contrasting motivations of seeking care and, simultaneously, shelter from a threatening caregiver. Therefore, this dimension represents a particular and delicate case. On the other hand, avoidance and ambivalence have been connected to the adequacy of the care received (Ainsworth et al., 1978; De Wolff and van Ijzendoorn, 1997). When such care is inadequate, attachment is *deactivated*, in the avoidant case, or *hyperactivated*, in the ambivalent case, which is reflected in corresponding behaviors and internal states as explained next (Ainsworth et al., 1978; Parkes et al., 1993; Mikulincer et al., 2003; Mikulincer and Shaver, 2016):

(1) *Avoidance*. The avoidant child deactivates attachment and is, consequently, considered to be “cold” or “unemotional.” In particular, the child appears unemotional in the SSP and does not seek comfort in the caregiver, instead, they typically focus on exploration. More specifically, the avoidant child *“Focuses on toys or environment, and away from parent, whether present, departing, or returning. Explores toys, objects, and room throughout the procedure. Fails to cry on separation from parent. Actively avoids and ignores parent on reunion (i.e., by moving away, turning away, or leaning out of arms when picked up). Little or no proximity or contact seeking, distress, or expression of anger. Response to parent appears unemotional. Focuses on toys or environment throughout procedure.”* (Hesse, 2008, p. 569). Therefore, the characteristics that can be considered to represent an avoidant child are low activation of attachment – that we will refer to as *low need* to receive care – and high rates of exploration (low rates of attachment).
(2) *Ambivalence*. The ambivalent child hyperactivates attachment and is, consequently, considered to be “hyper-emotional.” In particular, during the SSP, the child appears worried about the caregiver’s availability and continuously seeks their presence – they typically not only focus on the caregiver but easily feel unattended to and protest. More specifically, the ambivalent child *“Focuses on parent throughout much or all of procedure; little or no focus on toys or environment. May be wary or distressed even prior to separation. Preoccupied with parent throughout procedure; may seem angry or passive. Fails to settle and take comfort in parent on reunion, and usually continues to focus on parent and cry. Signs of anger toward parent are mixed with efforts to make contact, or are markedly weak. Fails to return to exploration after reunion, as well as during separation and often preseparation as well (i.e., preoccupied by parent, does not explore).”* (Hesse, 2008, p. 569). Therefore, the characteristics that can be considered to represent an ambivalent child are high activation of attachment – that we will refer to as *high need* to receive care – and high rates of attachment (low rates of exploration).

For each dimension, the acquired representations are primarily implicit (non-verbal) and deducible from the manifested patterns. In this case, the above descriptions – which are supported by expert ratings of a very large SSP sample (Fraley and Spieker, 2003) and by objective measurements on video and audio recordings (Chow et al., 2018; Prince et al., 2021) – suggest that: (1) Avoidant representations concern the caregiver’s emotional connection; and (2) Ambivalent representations concern the caregiver’s physical attendance.

##### Caregiving features

Although the way in which attachment dimensions derive from the features of caregiving (intergenerational transmission) is still controversial in attachment theory (Verhage et al., 2016; van Ijzendoorn and Bakermans-Kranenburg, 2019), the dimensionality of attachment suggests a possible corresponding dimensionality of caregiving (Harrist and Waugh, 2002; Skinner et al., 2005; Bernier et al., 2014; Feldman, 2017; Hollenstein et al., 2017; Gagliardi, 2021). In other words, if several attachment dimensions can be identified as characterizing the child (or any “attacher”), some corresponding caregiving features should be identified as characterizing the mother (or any caregiver). Our model implements this hypothesis, focusing on the two dimensions of avoidance and ambivalence. In particular, consistently with the above descriptions, it considers avoidance as induced by the caregiver’s *insensitivity* (an emotional feature) and ambivalence as induced by the caregiver’s *unresponsiveness* (a physical feature; Gagliardi, 2021, 2022):

(1) *Insensitivity*. Avoidance is induced by an insensitive caregiver, who does not activate their caregiving system when the child would need them to be sensitive (i.e., emotionally connected). As a result, the child stops activating their attachment system: If the caregiver seems not to care (emotionally disconnected), this will discourage the child from asking for care.
(2) *Unresponsiveness*. Ambivalence is induced by an unresponsive caregiver, who does not activate their caregiving system when the child would need them to be available (i.e., physically attendant). As a result, the child insists on activating their attachment system: If the caregiver seems to be often “distracted by other matters” (physically non-attendant), the child will be more persistent in reminding the caregiver to be available.

Therefore, the two dimensions can be conceptualized as follows (Gagliardi, 2021):

1. Avoidance has an “*emotional*” nature, meaning that it concerns the emotional connection the caregiver offers to the child. The sensitive caregiver is emotionally connective, and when the child needs emotional care (e.g., “*I’m feeling lonely*”), they are ready to offer it. If the caregiver does not provide emotional comfort (i.e., they are insensitive), then the child feels there is no point in asking for it. They tend to deactivate attachment and become avoidant (Mikulincer et al., 2003).
2. Ambivalence has a “*physical*” nature, meaning that it concerns the caregiver’s availability. The responsive caregiver is available when the child feels the caregiver should be there for them (e.g., “*Hey, where are you?*”). If the caregiver cannot attend (i.e., they are unresponsive), then the child feels that increasing their requests should catch the caregiver’s attention. They tend to hyper-activate attachment and become ambivalent (Mikulincer et al., 2003).

Emotional and physical components are involved in both dimensions. But to stress their origin and distinction, we refer to avoidance as the emotional dimension and to ambivalence as the physical one. Any combination of the two dimensions is possible.

##### Trends

**FIGURE 1:**
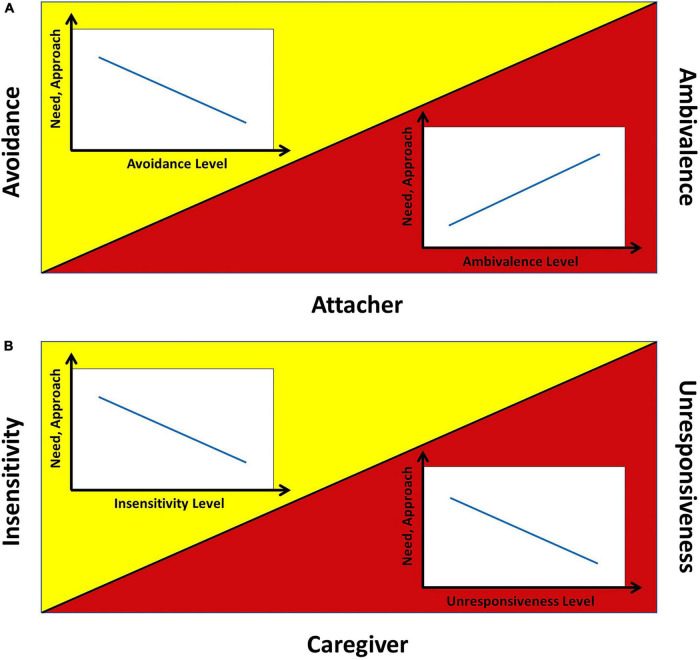
Expected trends of attacher’s and caregiver’s need and approach. Following the literature, this figure represents indicative linear trends of need and approach: **(A)** in the attacher case, for avoidance (yellow side) and ambivalence (red side) and **(B)** in the caregiver case, for insensitivity (yellow side) and unresponsiveness (red side). Exploration always has the opposite trend.

According to the above discussion, the literature suggests that the child’s and caregiver’s expected need, approach, and exploration have monotonic trends for increasing levels of avoidance or ambivalence. Assuming indicative linear trends, we can graphically represent the expected trends in the avoidant and ambivalent cases as shown in Figure 1.

##### Targets

The trends of behavior and internal states of the avoidant and ambivalent dyads suggest that child and mother have different targets (i.e., different set-goals) according to their dimensional level. In other words, one’s representation of the relationship related to a given dimension sets the goal they pursue while interacting. More specifically: (1) The avoidant child and insensitive caregiver have the same representational targets of emotional connection: the higher the avoidance and insensitivity, the lower the connection (less need, less approach); (2) The ambivalent child and unresponsive caregiver have contrasting representational targets of (psychological) distance (for physical attendance): the higher the ambivalence and unresponsiveness, the lower the distance pursued by the child (more need, more approach), the higher the distance pursued by the caregiver (less need, less approach).

Summarizing, according to the DP: (1) the two dimensions avoidance and ambivalence correspond to the two caregiving features insensitivity and unresponsiveness, respectively; (2) Behaviors and internal states have characteristic trends (3) that correspond to specific dimensional targets. Our model will need to account for all these aspects of the relationship.

## The dimensional attachment model: A two-dimensional agent-based model of attachment

The *Dimensional Attachment Model* (DAM) is an agent-based model (ABM) with two agents – an attacher and a caregiver. Although it can potentially represent any dyad, since some parameters that describe the agents need to be particularized, the prototypical child-mother case is considered here. In the model, an environment populated by the two agents is iteratively simulated, making their attachment-relevant variables change according to rules compliant with the DP.

In developing the DAM, our goal was to test if a child and mother that behave according to the DP generate the expected avoidant and ambivalent patterns (as described above) in terms of both internal states and behavior. With this purpose, we built a discrete model directly inspired by the psychological theory. However, given the nature of the model, we give a mathematical description compatible with dynamical systems theory (Thelen and Smith, 1994; Port and van Gelder, 1995), where representations are generally described as state variables or control parameters. For example, psychologically, the drives for attachment and caregiving (*a* and *c* below) are representations of motivational states, and the ambivalence and avoidance stored values (*A*_*v*_ and *A*_*m*_) are representations of the (cognitive, emotional, and sensorimotor) internal states learned for those dimensions. In the description of the model, the former (*a* and *c*) are variables, and the latter (*A*_*v*_ and *A*_*m*_) parameters. Through simulation, we explore the effects of different values of the parameters on the observed dynamics of the system as described by the variables.

Each iteration step, *n*, marks a psychological event (such as taking care of the child) and, therefore, iterations beat a “psychological time,” in other words, from one iteration to the other, the elapsed time can be different (for example, the time spent taking care of the child can be different in different interactions).

As previously noted, child and mother each have two intrinsic motivations. The child is motivated by the attachment motivational system – that they direct toward the mother – and, coherently, the mother is motivated by the caregiving motivational system – that she directs toward the child. Both agents also have an exploration motivational system. Active motivations are expressed behaviorally through position changes: Attachment by approaching the mother; Caregiving by approaching the child; Exploring by moving toward an object of interest (or in a random direction if no such object is detected).

**FIGURE 2:**
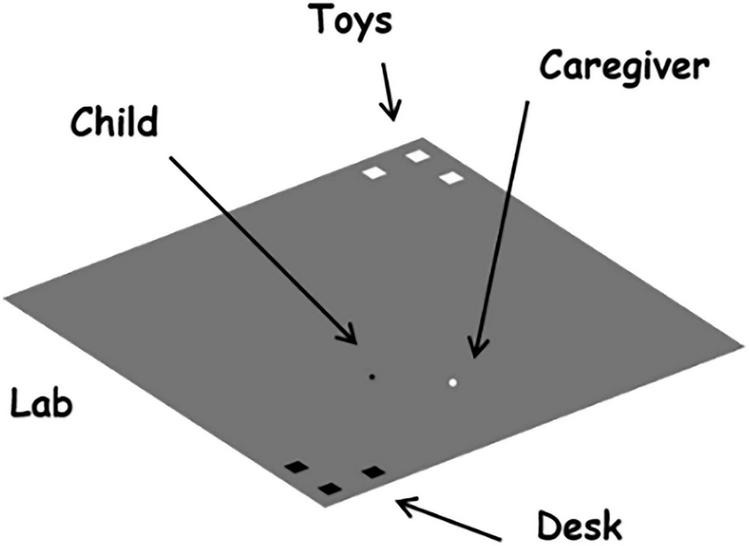
The agents and the simulation environment. The lab (simulation environment) resembles a large square room, where a child (black dot) and a caregiver (white dot) are free to move. The lab has some objects of interest for the child (white squares at the top corner; e.g., toys) and some objects of interest for the caregiver (black squares at the bottom corner; e.g., a desk).

The simulation environment is a 2D square “lab,” intended to resemble a typical SSP setting, that is empty except for the presence of a few objects in two opposite corners: objects of interest for the child in the top corner and objects of interest for the mother in the bottom corner (Figure 2). The asymmetric relationship between child and mother is represented in terms of “speed” – the maximum distance that an agent can cover from an iteration to the other – and “vision” – the distance from which an agent can detect an object interesting for them – by giving the caregiver three times the speed and vision of the child.

### The rationale of the model

As discussed above, attachment has an evolutionary function, and its dimensions express the adaptation to corresponding caregiving features (Bowlby, 1969/1982; Chisholm and Sieff, 2014; Simpson and Belsky, 2016; Szepsenwol and Simpson, 2019; Gagliardi, 2021). In other words, the dimensionality of attachment suggests the independent acquisition of each dimensional level from the detection of a specific caregiving feature. In particular, we assume the independent acquisition of avoidant and ambivalent levels from the caregiver’s insensitivity and unresponsiveness, respectively.

Given their evolutionary role, each dimension will be elicited by a context recognized as having the corresponding adaptive value. For example, when the child will focus on signals related to emotional care (a loving look of the caregiver, for example), the avoidant dimension will come into play (and the child may respond with a happy smile). Therefore, although simultaneous elicitation of multiple dimensions cannot be excluded, it can reasonably be assumed that, in any given interaction session, only one dimension will be elicited. This is especially true of avoidance and ambivalence as they cannot be expressed simultaneously because they entail attachment deactivation and hyper-activation, respectively (Mikulincer et al., 2003; Mikulincer and Shaver, 2016). Taking this into account, our attachment model implements the two dimensions separately and selects one of them to be expressed in each simulation run.

**FIGURE 3:**
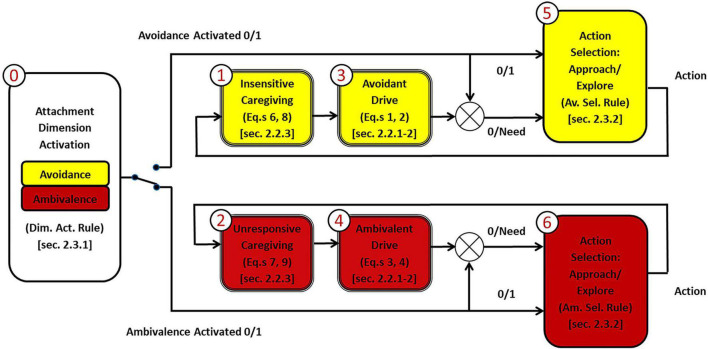
The rationale of the model. The attacher activates a dimension, and corresponding interactions take place. The activation of avoidance or ambivalence determines the generation of avoidant or ambivalent actions, which push the attacher toward the set-goal corresponding to the (stored) level of the activated dimension.

We provide here a functional diagram representing the rationale of our model (Figure 3), describing each of its components and their relation to the model implementation (each block corresponds to an implementation section below).

Adopting the child’s perspective, as a preliminary step before the beginning of the simulation session (block 0), the dimension that determines the following interactions’ type is activated. Such interactions will be either avoidant (upper branch of the diagram) or ambivalent (lower branch of the diagram). An activation mechanism depending on the child’s dimensional levels could be considered (dimension activation rule below). In this case, an avoidant child will be highly sensitive to the caregiver’s insensitivity and tend to activate avoidance, while an ambivalent child will be highly sensitive to the caregiver’s unresponsiveness and tend to activate ambivalence. If avoidance is selected (switch toggled in upper position), the caregiving context is recognized as (in)sensitive (block 1) by focusing on the caregiver’s exploration rate (Equations 6, 8). Then a non-zero avoidant drive (block 3; Equations 1, 2) is calculated, a need is delivered to the avoidant action selection system (block 5; avoidant selection rule below), and an avoidant action is generated. On the other hand, if ambivalence is selected (switch toggled in lower position), the caregiving context is recognized as (un)responsive (block 2) by focusing on the distance of the caregiver (Equations 7, 9). Then a non-zero ambivalent drive (block 4; Equations 3, 4) is calculated, a need is delivered to the ambivalent action selection system (block 6; ambivalent selection rule below), and an ambivalent action is generated. In both cases, the action produced will be either an approach to the caregiver (attachment) or an explorative move. This action will tend to make the next child’s dimensional level (i.e., representation) closer to the stored one (set-goal).

To further clarify, the attachment interactions expressed by our model can be described as follows. Once a dimension is selected (block 0) and the simulation starts, at each iteration:

1. The child builds a current perception of dimensional level (i.e., a representation) from the caregiver’s behavior. More specifically: In case of avoidance, the mother’s exploration rate (behavioral variable) will affect the child’s “emotional separation” (psychological variable; block 1); In case of ambivalence, the mother’s distance (behavioral variable) will affect the child’s “perceived distance” (psychological variable; block 2).
2. This current dimensional level and the other relevant variables and parameters induce some need for care in the child: Need for emotional care in case of avoidance (block 3); Need for physical care in case of ambivalence (block 4).
3. Finally, the child compares their current perception of dimensional level to their target one and takes an action – depending on the need level – that tends to make the next perception closer to the target. In other words: In case of avoidance, the emotional separation felt by the child will tend to their avoidant target (block 5); In case of ambivalence, the distance perceived by the child will tend to their ambivalent target (block 6). Attachment works as a control system with dimensional (i.e., representational) set-goals.

The caregiver behaves similarly, expressing psychological variables that are consistent with their own behavioral ones.

#### Model description

The overall system (Figure 3) can conveniently be thought of as consisting of a core (blocks 1–4) and an interface (blocks 0 and 5–6), through which it interacts with the environment. Below, we describe these parts in turn. As done above, we primarily refer to the attachment system, which is the focus of this work (similar considerations hold for the caregiving system).

### The attachment system’s core

We first describe the core elements of our model as expressed in blocks 1–4 of Figure 3. Since the drives specify the different components involved in the activation of the attachment and caregiving systems, we start with them. We want to stress that we use the terms “drive” and “need” to refer to key variables without implying that these correspond to classical notions of drive and need in the literature on human motivation (see e.g., Cofer and Appley, 1964).

#### Drives

A drive is defined as what generates a need (i.e., the system’s activation) by combining the multiple factors involved. Between them, the time passed without providing care and what child and caregiver signal to each other have been documented as essential elements of the attachment relationship (Bowlby, 1973; Ainsworth et al., 1978). Following the DP-informed theory discussed above, for the formulation of the drives (Equations 1–4), we consider that, other things being equal:

1. The avoidant child (Equation 1) will feel a greater drive to receive care when: (i) its avoidance level (*A*_*v*_) is smaller; (ii) the time with no emotional care (*K*) is longer; (iii) the need to provide care signaled by the caregiver (*N*_*G*_) is smaller; and (iv) the perceived emotional separation (*S*_*E*_) is greater. (A similar consideration holds for the insensitive caregiver in Equation 2.)
2. The ambivalent child (Equation 3) will feel a greater drive to receive care when: (i) its ambivalence level (*A*_*m*_) is greater; (ii) the time with no physical care (*K*) is longer; (iii) the need to provide care signaled by the caregiver (*N*_*G*_) is greater^2^ ; and (iv) the perceived distance (*D*_*P*_) is greater (i.e., less availability). (A similar consideration holds for the unresponsive caregiver in Equation 4.)

Consistently, two pairs of coupled equations are proposed for the activation of attacher avoidance, drive *a*_*av*_, and caregiver insensitivity, drive *c*_*av*_ (block 3):


(1) $$a_{a\,v}\left[n+1\right]=\left(1-A_{v}\right)\left(K_{a\,v}\left[n\right]/2\right)+C_{f,a\,v}(1-N_{G,a\,v}$$

$$\left(c_{a\,v}\left[n\right],A_{v}\right))S{}_{Ea}[n]+c{}_{0\,a,a\,v}$$

(2) $$c_{a\,v}\left[n+1\right]=\left(1-I_{n}\right)K_{a\,v}\left[n\right]/2+C_{f,a\,v}(1-N_{R,a\,v}$$

$$\left(a_{a\,v}\left[n\right],I_{n}\right))S{}_{E\,c}[n]+c{}_{0\,c,a\,v}$$

And two pairs of coupled equations are proposed for the activation of attacher ambivalence, drive *a*_*am*_, and caregiver unresponsiveness, drive *c*_*am*_ (block 4):


(3) $$a_{a\,m}\,\left[n+1\right]=A_{m}\,K_{a\,m}\,\left[n\right]/2+C_{f,a\,m}\,N_{G,a\,m}$$

$$\left(c_{a\,m}\,\left[n\right],1-A_{m}\right)\,D_{P\,a}\,\left[n\right]+c_{0\,a,a\,m}$$

(4) $$c_{a\,m}\,\left[n+1\right]=\left(1-U_{n}\right)\,K_{a\,m}\,\left[n\right]/2+C_{f,a\,m}$$

$$\left(1-N_{R,a\,m}\,\left(a_{a\,m}\,\left[n\right],U_{n}\right)\right)\,D_{P\,c}\,\left[n\right]+c_{0\,c,a\,m}$$

In these equations: (1) *K* is a measure of the elapsed psychological time since the child last received care; (2) *N* is the need signalled by the other agent that they require care (*N*_*R*_), or wish to express caregiving (*N*_*G*_); (3) *S*_*E*_ is a measure of the “emotional separation” experienced by both agents; (4) *D*_*P*_ is the “perceived distance” between the agents. Each of these elements are explained in more detail in the following subsections. (5) *A*_*v*_ is the level of the attacher’s avoidance, and *I*_*n*_ is the level of the caregiver’s insensitivity, while (6) *A*_*m*_ is the level of the attacher’s ambivalence, and *U*_*n*_ is the level of the caregiver’s unresponsiveness. These last four are control parameters set at the start and maintained fixed throughout the simulation run. *A*_*v*_ and *A*_*m*_ represent the dimensional levels stored in the attacher’s brain. (7) *C*_*f,av*_ and *C*_*f,am*_ are coupling factors, which determine the weight of each agent’s need on the other. (8) *c*_*0a,av*_, *c*_*0c,av*_, *c*_*0a,am*_, and *c*_*0c,am*_ are constants used for the initial setting of the system.

#### Needs and elapsed time since care

**FIGURE 4:**
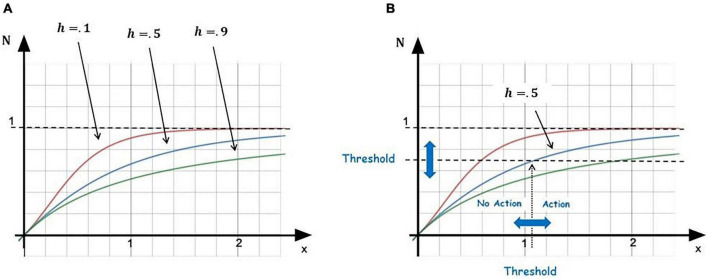
Calculation of the need function, *N*. **(A)** Three different levels of parameter *h* are shown (0.1, 0.5, 0.9) to illustrate that an increasing *h* reduces the steepness of the curve. **(B)** A threshold is set so that, when *N* is greater than the threshold, the agent can perform an attachment or caregiving behavior. Here, the case of *h* = 0.5 and corresponding threshold is shown.

The drives generate a need according to the function:


(5) $$N\,\left(x,h\right)=\frac{x}{\left(x+h^{x}\right)}$$

where the variable *x* is the relevant drive (*a* or *c*) and the parameter *h* accounts for the dimension level (*A*_*v*_ or *A*_*m*_), which equals the corresponding feature level (*I*_*n*_ or *U*_*n*_; Figure 4). This need function expresses the child’s need to receive care (*N*_*R*_, activation level of their attachment system) and the mother’s need to give care (*N*_*G*_, activation level of her caregiving system). It is assumed that each agent can perceive the other’s need level, and two pairs of Equations 1–2 and 3–4, are copuled in this way. *N*(*x*,*h*) has the form of a Hill function (Somvanshi and Venkatesh, 2013), commonly used to model saturation in biological systems, and is parameterized by *h* such that the steepness of the curve reduces with increasing *h* (Figure 4A). This reflects, for example, the fact that the more a child is avoidant (larger *h*), the less they feel a change in the need to be taken care of (for a given change of the situation). A phenomenon that is well represented by the avoidant child reaction to a separation in the SSP.

*K* is the time passed with no provision of care, which relates to emotional care in the case of avoidance (*K*_*av*_) and to physical care in the case of ambivalence (*K*_*am*_). At each interaction *n*, this is equal to the number of iterations since care was last provided, considering care as provided when *N*_*G*_ exceeds its threshold. When *K* becomes zero, the need function *N* drops.^3^ The coefficient 1/2 of *K* was set empirically and could be changed to account for environmental variations.

**FIGURE 5:**
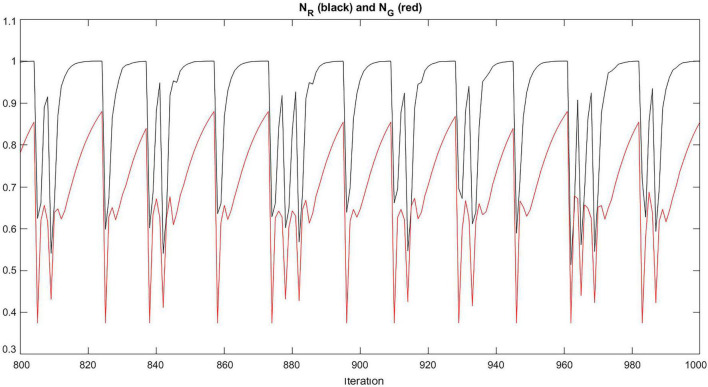
Oscillation of *N*_*R*_ and *N*_*G*_. For each dimension, *N*_*R*_ and *N*_*G*_ oscillate around a baseline. The graph represents the oscillation of *N*_*R*_ (in black) and *N*_*G*_ (in red) for ambivalence 0.7 over 200 iterations.

The modeled interaction between child and caregiver corresponds to the oscillation of the drives, *a* and *c*, and the needs, *N*_*R*_ and *N*_*G*_, around a baseline as illustrated in Figure 5 for an example simulation run. The need oscillations will generate a behavioral dynamics of alternating approach and exploration, with rates that depend on the level of avoidance or ambivalence (cf. Simulations section).

#### Perceptions of emotional and physical distance

At each iteration, the agents experience an “emotional separation,” *S*_*E*_, and “perceived distance,” *D*_*P*_, connected to contextual cues. More specifically: (1) In the avoidant case, the attacher experiences *S*_*Ea*_ and the caregiver *S*_*Ec*_; (2) In the ambivalent case, the attacher experiences *D*_*Pa*_ and the caregiver *D*_*Pc*_. The use of different variables is due to the different nature of the two dimensions and their link to different contextual cues, as discussed next.

Following the DP, the terms “emotional separation” and “perceived distance” reflect the assumption that avoidance is an emotional dimension and ambivalence is a physical dimension. *S*_*E*_ refers to the emotional connection and *D*_*P*_ to the physical availability perceived by the child in the relationship. These “psychological variables” are connected to “behavioral variables” measurable in the lab. In particular, for each dimension, a variable related to the caregiver’s behavior provides a cue to the child to derive a dimensional level corresponding to the current situation. The child will compare this level with the target one stored in their mind to drive their action. In this perspective, attachment works as a multidimensional control system.

To derive *S*_*E*_ and *D*_*P*_, we used the following behavioral variables:

1. “indifference” (*i*): defined as the percentage of iterations in which the caregiver explores, where *N*_*ex*_ is the number of such explorations:

(6) $$i\left[n\right]=\frac{100\;N_{ex}\left[n\right]}{n}$$

^4^

2. “distancing” (*d*): defined as the distance between child and caregiver, where (*x*_*a*_,*y*_*a*_) and (*x*_*c*_,*y*_*c*_) are the positions in the lab of the attacher and the caregiver, respectively:

(7) $$d\,\left[n\right]=\sqrt{{\left(x_{a}\,\left[n\right]-x_{c}\,\left[n\right]\right)}^{2}+{\left(y_{a}\,\left[n\right]-y_{c}\,\left[n\right]\right)}^{2}}$$

From them, each agent obtains *S*_*E*_ and *D*_*P*_ through an update rule of the form:


C⁢u⁢r⁢r⁢e⁢n⁢t⁢P⁢e⁢r⁢c⁢e⁢p⁢t⁢i⁢o⁢n=P⁢r⁢e⁢v⁢i⁢o⁢u⁢s⁢P⁢e⁢r⁢c⁢e⁢p⁢t⁢i⁢o⁢n+S⁢t⁢e⁢p⁢(O⁢b⁢s⁢e⁢r⁢v⁢e⁢d⁢ D⁢e⁢v⁢i⁢a⁢t⁢i⁢o⁢n-P⁢r⁢e⁢v⁢i⁢o⁢u⁢s⁢D⁢e⁢v⁢i⁢a⁢t⁢i⁢o⁢n),

with a noisy step size representing the natural uncertainty of the agent’s perception. The particular expressions used are:


(8) $$S_{E}\,\left[n\right]=S_{E}\,\left[n-1\right]+2\,r\,\left[\left(i\,\left[n\right]-T_{i}\right)-\left(S_{E}\,\left[n-1\right]-T_{E}\right)\right]$$

(9) $$D_{P}\,\left[n\right]=D_{P}\,\left[n-1\right]+2\,r\,\left[\left(d\,\left[n\right]-T_{d}\right)-\left(D_{P}\,\left[n-1\right]-T_{P}\right)\right],$$

which update the previous values (first term) depending on the current indifference or distancing (second term), thereby going from observable variables (*i*, *d*) to mental ones (*S*_*E*_, *D*_*P*_) – as suggested by the DP. In these equations, *r* ∈ [0,1] is a uniformly distributed random number, *T*_*E*_, *T*_*i*_, *T*_*P*_, and *T*_*d*_ are the target values of *S*_*E*_, *i*, *D*_*P*_ and *d*, respectively (as discussed below). The effectiveness of this formula can be clarified considering the following. The update needs to depend on the targets: for a new dimensional level to be adequate, it has to be consistent with the corresponding target. By referring the current behavioral gap from target (*i*[*n*]−*T*_*i*_ or *d*[*n*]−*T*_*d*_) to the previous psychological gap from target (*S*_*E*_[*n*−1]−*T*_*E*_ or *D*_*P*_[*n*−1]−*T*_*P*_), this expression ensures an adequate update. For example, considering the distance (Equation 9), if the new *d* is further from its target than the old *D*_*P*_ from its, then it makes sense that the new *D*_*P*_ increases. If *d* is closer, it makes sense that *D*_*P*_ decreases. The behavioral variable provides a consistent update of the psychological one.

### The attachment system’s interface

To describe how the system interacts with the environment requires the specification of blocks 0 and 5–6 of Figure 3, which correspond to the dimension activation and action selection rules. These are essential to close the loop through the environment via perception and behavior. We start examining action selection, which follows the above-described processing of drives and needs (blocks 1–4). The dimension activation was not an object of our implementation, but we suggest how it could be done at the end.

#### Action selection and behavior expression

**FIGURE 6:**
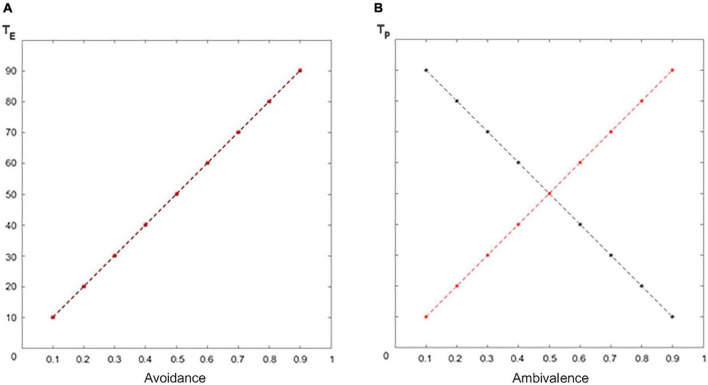
Dimensional targets. **(A)** Emotional separation and **(B)** perceived distance targets (black for the attacher, red for the caregiver).

For each agent, the system compares the current dimensional level to the target (stored) one and takes an action that tends to decrease the difference between the two. A decision is made according to the threshold that characterizes the agent’s need *N*. Specifically, when *N_R_* exceeds its threshold (*T_R_*), the attacher needs care, and when *N_G_* exceeds its threshold (*T_G_*), the caregiver needs to provide care (Figure 4B). The thresholds are given by the following expression:


(9) $$T=T_{b\,l}\pm \tau \,\left(1+r\right)$$

where, *T_bl_* is a baseline value, τ is a constant, and *r* ∈ [0,1] is a uniformly distributed random number (to account for possible fluctuations, given that *T* is a subjective/psychological variable). *T* is reduced (minus sign in the formula) when *N* decreases. This is intended to model the prudential tendency to readily reactivate attachment or caregiving when they are deactivated, as expected given their role for contingent survival. The constants were set empirically (cf. Simulations section). As discussed above, according to the DP, the avoidant and ambivalent dyads differ for the goals they set for themselves. Therefore, the following is considered:

1. In the avoidant case, the agents have the same goals in terms of emotional separation (*T*_*E*_). The more an agent is avoidant/insensitive (0.1–0.9), the larger the emotional separation they want to keep. In our model: *T*_*E*_ = 100*A*_*v*_ = 100*I*_*n*_ (10–90; Figure 6A).
2. In the ambivalent case, the agents have opposite goals in terms of perceived distance (*T*_*P*_). The more the attacher is ambivalent (0.1–0.9), the smaller the perceived distance they want to keep. The more the caregiver is unresponsive (0.1–0.9), the larger the perceived distance they want to keep. In our model: *T*_*P*_ = 100(1−*A*_*m*_) (90–10) for the attacher and *T_P_=100U_n_* for the caregiver (10–90; Figure 6B).

The target emotional separation (*T*_*E*_) and perceived distance (*T*_*P*_), respectively, represent the psychological values of emotional separation (*S*_*E*_) and perceived distance (*D*_*P*_) that maximize the subject’s comfort. For the child and the caregiver, such targets vary according to the level of avoidance or ambivalence.

In general, the targets in the mind of the agents (*T*_*E*_, *T*_*P*_) will correspond to targets observable in the context of interaction (*T*_*i*_, *T*_*d*_). In the case of our elementary squared environment, we used the simple linear relationships *T*_*i*_ = 1.1*T*_*E*_ (for the avoidant attacher and insensitive caregiver) and *T*_*d*_ = 0.24*T*_*P*_ (for the ambivalent and unresponsive caregiver).^5^

The action selection mechanism is implemented for the movement in the lab based on the agents’ needs and targets. The child will decide whether to approach – a manifestation of the need to receive care, i.e., attachment – or explore. The caregiver will decide whether to approach – a manifestation of the need to provide care to the child, i.e., caregiving – or explore. For each agent, approaching is a movement toward the other agent, while exploring is a movement toward an object of interest or random (when no object is found). Each move is a change in position that cannot exceed the agent’s speed.^6^

**FIGURE 7:**
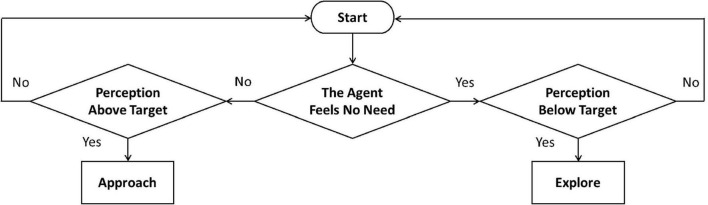
Action selection rule. An action is selected depending on the comparison between current and target dimensional levels.

Given the need *N* and its threshold *T*, the implemented decision rule is (Figure 7):

1. if *N* < *T* (the agent “feels no need”), if *S*_*E*_ < *k*_*E*_*T*_*E*_ (in the avoidant case)/*D*_*P*_ < *k*_*P*_*T*_*P*_ (in the ambivalent case), then explore;
2. if *N* > *T* (the agent “feels a need”), if *S*_*E*_ > *k*_*E*_*T*_*E*_ (in the avoidant case) / *D*_*P*_ > *k*_*P*_*T*_*P*_ (in the ambivalent case), then approach.

Need is need to receive care in the case of the child and need to provide care in the case of the caregiver; *S*_*E*_ is compared in the avoidant case, *D*_*P*_ is compared in the ambivalent case; *T*_*E*_ and *T*_*P*_ are, respectively, the target emotional separation and perceived distance for the agent; *k*_*E*_ and *k*_*P*_ are constant values (cf. Simulations section).

The system acts as a multi-dimensional controller. It compares current dimensional levels to target ones and takes actions that tend to decrease the difference between the two.

##### Approach and exploration

An agent’s travel toward a target, i.e., the other agent (approach) or an object of interest (exploration), can be described as follows:


x⁢[n+1]=x⁢[n]+△⁢x⁢[n]

y⁢[n+1]=y⁢[n]+△⁢y⁢[n]

When the target’s position (*x*_*t*_,*y*_*t*_) is beyond the agent’s speed limit, the update is calculated according to such a limit and the angle identified by the target:


△⁢x⁢[n]=s⁢p⁢e⁢e⁢d⋅cos⁢(a⁢n⁢g⁢l⁢e)

△⁢y⁢[n]=s⁢p⁢e⁢e⁢d⋅s⁢i⁢n⁢(a⁢n⁢g⁢l⁢e)

where, $a\,n\,g\,l\,e=\cos^{-1}\left(\sqrt{{\left(x-x_{t}\right)}^{2}}/d_{t}\right)=\sin^{-1}\left(\sqrt{{\left(y-y_{t}\right)}^{2}}/d_{t}\right)$, *d*_*t*_ distance to the target. When the target is below the speed limit, the agent moves to a random position whose coordinates differ no more than 0.5 from those of the target. If the agent wants to explore and objects of interest are in sight, exploration is made toward the nearest one. After an object has been explored, it loses its attraction for a certain number of iterations. If no interesting object is found, exploration is a move in a random direction.

#### Dimension activation

For any given simulation session, interactions can be either avoidant or ambivalent. A basic activation mechanism based only on the dimensional level could be implemented by a winner-take-all rule that evaluates each level’s softmax function (Bishop, 2006) and selects its maximum:

*d*_*i*_,*i* = 1,2 *selected when s*(*d*_*i*_) = *Max*(*s*(*d*_1_),*s*(*d*_2_)), *where:*

*d*_1_ = *A*_*v*_ *(avoidance)*, *d_2_=A_m_ (ambivalence)*,

$s\,\left(d_{i}\right)=\frac{e^{\beta \,\left(d_{i}+r_{i}\right)}}{\sum_{j=1,2}e^{\beta \,\left(d_{j}+r_{j}\right)}}$ *(softmax function)*,

*r*_1_,*r*_2_, *normally distributed random numbers.*

Here, the random numbers *r*_*i*_ account for contextual noise, and the parameter β can be used to act on the influence of the dimensional levels’ gap. A larger β tends to invert the effect of such a gap.

## Simulations

For all^7^ simulations, the lab size *S* was set to 30 (lab coordinates 1 to *S*, actual size *S-1*). Moreover, the following was chosen: (1) For the child: speed *L*/9 and vision *L*/3; (2) For the mother: speed *L*/3 and vision *L*/1; given $L=\sqrt{2}\,S$. Each agent has 3 objects of interest, which lose their status for 7 iterations after being explored.

The simulations of avoidant and ambivalent interactions were performed separately, considering 9 values for each dimension – *A*_*v*_ = 0.1, 0.2,…,0.9, *A*_*m*_ = 0.1, 0.2,…,0.9. A higher value corresponds to a stronger acquisition of the dimension. Constants values for the system were set as follows:

1. In Equations 1–2: *C*_*f*,*av*_ = 4, *c*_0*a*,*av*_ = 0.49, *c*_0*c*,*av*_ = 0.5;
2. In Equations 3–4: *C*_*f*,*am*_ = 2, *c*_0*a*,*am*_ = 0.2, *c*_0*c*,*am*_ = 0.5;
3. In Equation 10: *T_bl_* = 0.75, τ = 0.08;
4. In the action selection rule: *k*_*E*_ = 1.01, *k*_*P*_ = 1.1.

A sensitivity analysis demonstrates that coupling the equations this way improves the system’s performance (i.e., *C*_*f*,*av*_ = 4 vs. *C*_*f*,*av*_ = 0 and *C*_*f*,*am*_ = 2 vs. *C*_*f*,*am*_ = 0; cf. Appendix). Initial conditions were set equal in all simulations (*K* = 0, *N* = 0.75, *S_E_* = 50, *D_p_ = 50*, *i* = 55, *d* = 12). In particular, the agents start from the same given positions in the central part of the lab [child (9,15), mother (21,15)].

In each simulation, the agents are considered adapted to each other. In other words, the acquisition of the attachment dimensions in the child’s mind is assumed to have already been induced by the caregiver (*A_v_* = *I_n_*, *A_m_* = *U_n_*). The interactions that follow the dimensional acquisition are simulated, and the corresponding attachment patterns are assessed. Such patterns are expected to reproduce the quality of those outlined in attachment literature (as described above; (Ainsworth et al., 1978; Hesse, 2008), in terms of both internal states (need in our model) and behaviors (approach and exploration). In particular, while the avoidant child is relationship-independent (low in approach and high in exploration), the ambivalent child is relationship-dependent (high in approach and low in exploration). Although these characteristics may seem to belong to the same dimension, it will be shown how they are consistent with a two-dimensional phenomenon, as the DP suggests.

### Results

Simulations’ results are presented in terms of states and behaviors of the agents for different levels of attachment dimension. The case of avoidance (*A*_*v*_) and ambivalence (*A*_*m*_) are considered in turn. The attachment dimension is the only referred to since the corresponding caregiving feature [insensitivity (*I*_*n*_) or unresponsiveness (*U*_*n*_)] has the same value.

#### Behavioral patterns

**FIGURE 8:**
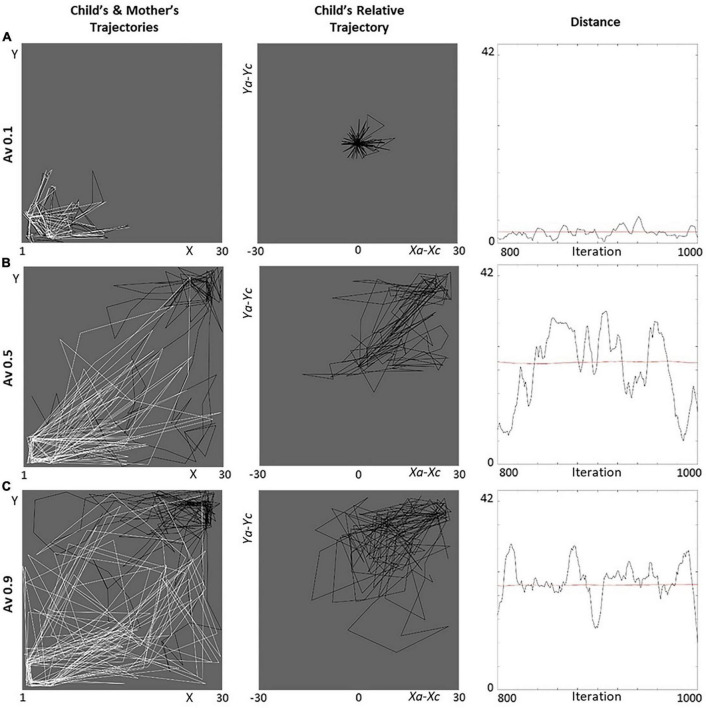
Behavior of avoidant dyads for different dimensional levels. Three avoidant levels represent the **(A)** “anti-avoidant” (*A*_*v*_ = 0.1), **(B)** secure (*A*_*v*_ = 0.5), and **(C)** (extremely) avoidant (*A*_*v*_ = 0.9) cases in terms of the agents’ trajectories (black for the child, white for the mother), child’s trajectory relative to mother, and distances (smoothed with a moving filter). In the left-column pictures, the objects of interest for child and mother are located in the top-right and bottom-left corners, respectively. All graphs refer to iterations 800–1,000 (the last 200 of our simulations).

**FIGURE 9:**
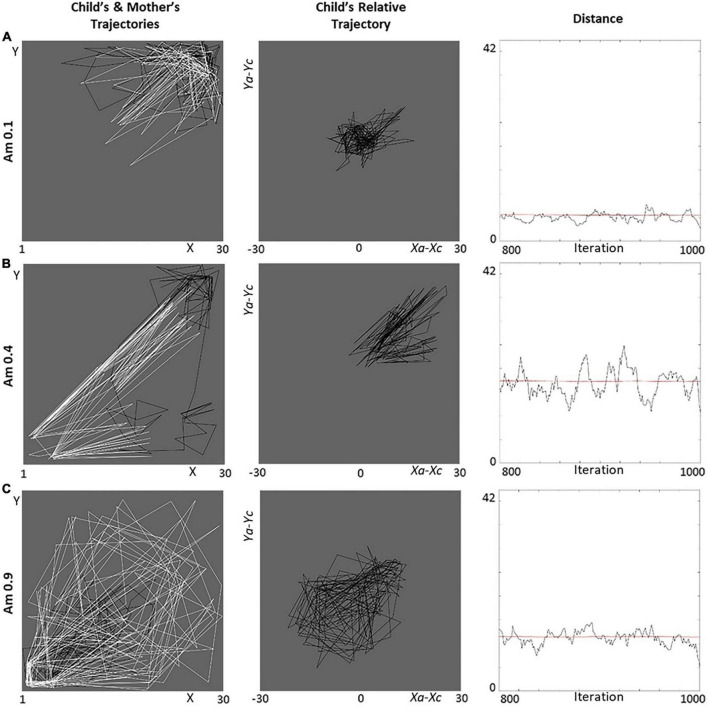
Behavior of ambivalent dyads for different dimensional levels. Three ambivalent levels represent the **(A)** ‘anti-ambivalent’ (*A*_*m*_ = 0.1), **(B)** secure (*A*_*m*_ = 0.4), and **(C)** (extremely) ambivalent (*A*_*m*_ = 0.9) cases in terms of the agents’ trajectories (black for the child, white for the mother), child’s trajectory relative to mother, and distances (smoothed with a moving filter). In the left-column pictures, the objects of interest for child and mother are located in the top-right and bottom-left corners, respectively. All graphs refer to iterations 800–1,000 (the last 200 of our simulations).

We first report relevant behavioral details concerning the simulations for representative levels of avoidance and ambivalence: (a) extremely low (*A*_*v*_ = 0.1, *A*_*m*_ = 0.1), (b) mid (*A*_*v*_ = 0.5, *A*_*m*_ = 0.4), and (c) extremely high (*A*_*v*_ = 0.9, *A*_*m*_ = 0.9; Figures 8, 9). We focus on the trajectories followed by the agents in the lab,^8^ the child’s trajectory relative to the caregiver ((*x*_*a*_−*x*_*c*_,*y*_*a*_−*y*_*c*_)), and the distance between the agents.

##### Avoidance (and insensitivity)

1. *A*_*v*_ = 0.1 (Figure 8A). The agents are “anti-avoidant” and manifest high activation of attachment and caregiving (need). As a result, they stick to each other (high approach, low exploration). Interestingly, they tend to gravitate around the objects of interest for the caregiver, who leads the interactions (Figure 8A-left). This pattern is emphasized by a very concentrated relative trajectory (Figure 8B-center) and low distances (Figure 8A-right).
2. *A*_*v*_ = 0.5 (Figure 8B). The agents appear secure, having an activation of attachment and caregiving (need) that results in a functional balance between approach and exploration. The child approaches moderately and tends to move around their objects of interest (exploration), while occasionally taken care of by the caregiver (Figure 8B-left). The appreciable proportion of exploration results in a relative trajectory toward the top-right corner (Figure 8B-center) and fairly high distances (Figure 8B-right).
3. *A*_*v*_ = 0.9 (Figure 8C). The agents appear (extremely) avoidant and manifest a very low activation of attachment and caregiving (need). As a result, they stick around their objects of interest or move randomly (exploration), and their trajectories are highly independent, as a sign of rare approach (Figure 8C-left). The autonomous exploration results in a spread relative trajectory (Figure 8C-center) and, again, relatively high distances (Figure 8C-right), which are, however, limited by the size of the lab and random moves.

##### Ambivalence (and unresponsiveness)

1. *A*_*m*_ = 0.1 (Figure 9A). The agents are “anti-ambivalent”: the child manifests high activation of exploration, and the caregiver of caregiving (need). As a result, the caregiver chases the child, and they tend to gravitate around the objects of interest for the child (Figure 9A-left). Consistently, the relative trajectory is very concentrated (Figure 9A-center), and distances are very little (Figure 9A-right).
2. *A*_*m*_ = 0.4 (Figure 9B). Similarly to the avoidant case (although with more approaches from the caregiver), the agents appear secure and have a functional activation of attachment and caregiving (need). The child again approaches moderately and tends to move around their objects of interest (exploration), while attended to by the caregiver (Figure 9B-left). The good proportion of exploration results in a relative trajectory on the right-top side (Figure 9B-center) and mid distances (Figure 9B-right).
3. *A*_*m*_ = 0.9 (Figure 9C). The agents appear (extremely) ambivalent: the child manifests very high activation of attachment, and the caregiver very low of caregiving (need). As a result, the child chases the caregiver, and the dyad tends to move around the caregiver’s objects of interest (Figure 9C-left). The exploration of the caregiver followed by the child makes the relative trajectory shift toward the bottom-left side (Figure 9C-center), and the high approach of the child limits the distances (Figure 9C-right).

##### The avoidant and ambivalent dyads in the lab

**FIGURE 10:**
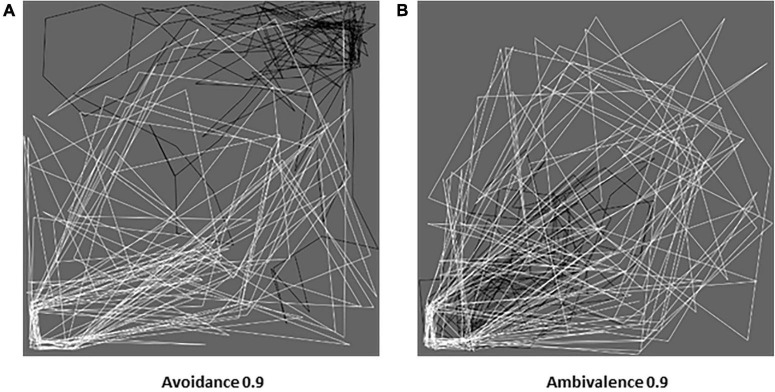
Avoidant and ambivalent dyads in action. Simulated trajectories (black for the child, white for the mother) followed by the extremely avoidant **(A)** and ambivalent **(B)** dyads (dimensional level 0.9, iterations 800–1,000). The comparison of the two pictures (extracted from Figures 8, 9) offers a glimpse of the different behavioral effects in the case of avoidant hyper-independence **(A)** and ambivalent hyper-dependence **(B)**.

Below (Figure 10), we compare the trajectories taken by the child (in black) and mother (in white) in the most avoidant (Figure 8C-left) and ambivalent (Figure 9C-left) cases. (a) The avoidant child and insensitive caregiver feel very little need (to receive and provide care, respectively) and move independently. Their paths concentrate where their objects of interest are located. (b) The ambivalent child feels very much in need (to receive care), while the unresponsive caregiver very little (to provide care). As a result, the child appears to insistently chase the caregiver, gravitating around the caregiver’s objects of interest. These patterns capture the essence of avoidance and ambivalence as described in the literature (cf. attached video clips^9^).

#### Mean values and trends

The percentage values over 1,000 iterations are reported for the following variables: (A) The need *N* – child’s need to receive care (*N*_*R*_) and caregiver’s need to give care (*N*_*G*_; above threshold). (B) Explorative and approaching behaviors. Also, the mean values over 1,000 iterations are reported for the distance between the two agents in the lab and the number of iterations with no provision of care.

The results obtained for avoidance/insensitivity and ambivalence/unresponsiveness are discussed considering the curves in their progression from left to right, i.e., for increasing dimensional values (black is used for the child, red for the mother).

##### Avoidance (and insensitivity)

**FIGURE 11:**
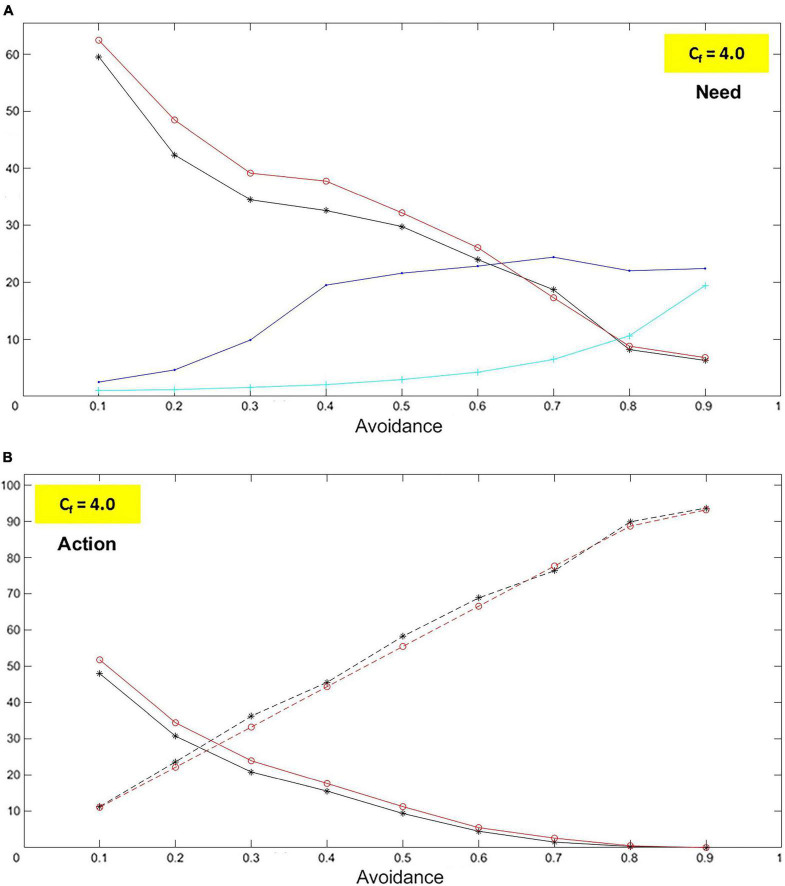
Avoidant case: Need and action. The graphs represent characteristics for the child (black curves) and the caregiver (red curves) for levels of avoidance (*A*_*v*_) and insensitivity (*I*_*n*_) ranging between 0.1 and 0.9 (with step 0.1). The blue curve represents the distance measured in the lab between the child and the caregiver. The cyan curve represents the number of iterations without caregiving. In particular, as *A*_*v*_ and *I*_*n*_ increase, it is shown that: **(A)** The needs to receive care (felt by the child) and give care (felt by the caregiver) decrease. **(B)** The child and the caregiver both increase their exploration (dashed curves) while they decrease their approaches. All these phenomena are entirely consistent with what attachment studies describe.

In the case of avoidance, the simulations produce a clear, and almost linear, decrease of both the need to receive care and the need to give care (Figure 11A). In other words: the more the child is avoidant, the less they need to be taken care of; the more the caregiver is insensitive, the less they need to provide care. In the less avoidant case, values are around 60% and, in the most avoidant one, just above 5%. Coherently, the simulations yield a sharp increase in exploration (dashed curves) and decrease in approaching (solid curves; Figure 11B). The former goes from a little over 10% to almost 95%, and the latter from about 50% to zero. These trends reflect what is expected from an avoidant dyad. Accordingly, the number of iterations with no provision of care rises (cyan curve; Figure 11A). On the other hand, the distance remains practically steady after a first increase, which can be explained by the limited size of the room where the agents move and random explorations (blue curve; Figure 11A).

##### Ambivalence (and unresponsiveness)

**FIGURE 12:**
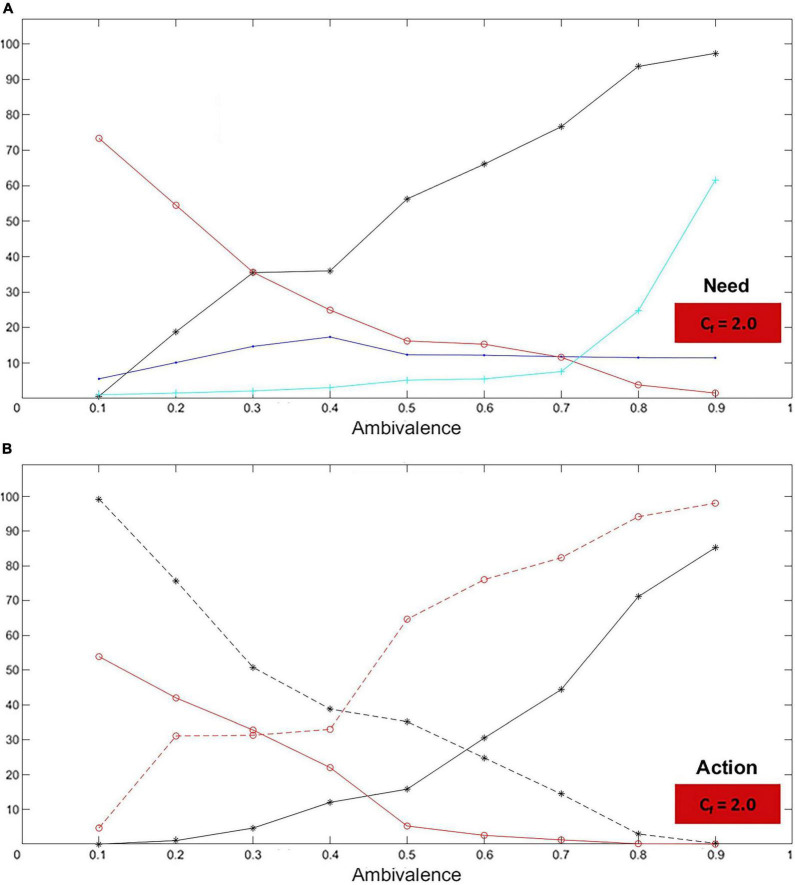
Ambivalent case: Need and action. The graphs represent characteristics for the child (black curves) and the caregiver (red curves) for levels of ambivalence (*A*_*m*_) and unresponsiveness (*U*_*n*_) ranging between 0.1 and 0.9 (with step 0.1). The blue curve represents the distance measured in the lab between the child and the caregiver. The cyan curve represents the number of iterations without caregiving. In particular, as *A*_*m*_ and *U*_*n*_ increase, it is shown that: **(A)** The need for care (felt by the child) increases while the need to give care (felt by the caregiver) decreases. **(B)** The child increases their approaches and decreases their exploration (dashed curve), while the caregiver increases their exploration (dashed curve) and decreases their approaches. All these phenomena are entirely consistent with what attachment studies describe.

In the case of ambivalence, the simulated needs to receive and give care have opposite trends: while the former increases sharply, the latter decreases (Figure 12A). The most non-ambivalent children show no need for care. Such a need rises and keeps soaring toward the most ambivalent case – to almost 100%. On the other hand, from the extremely responsive caregiver to the extremely unresponsive one, the decline in the need to give care is less wide – roughly, from a little above 70% to practically zero. Explorations and approaches are coherent with the needs (Figure 12B). The more the child becomes ambivalent, the more they approach the caregiver and the less they explore. Conversely, the more the caregiver becomes unresponsive, the more they explore and the less they approach the child. These trends match those expected from an ambivalent dyad. Accordingly, the number of iterations in which the caregiver is unresponsive becomes higher as the child becomes more ambivalent (cyan curve; Figure 12A). Interestingly, the distance between the agents seems to remain quite stable despite the significant change of the agents’ attitudes, which indicates that such attitudes compensate each other in terms of distance (blue curve; Figure 12A). In fact, the simulation of the most ambivalent case shows that the child constantly chases the caregiver.

#### Simulated vs. expected dynamics

**FIGURE 13:**
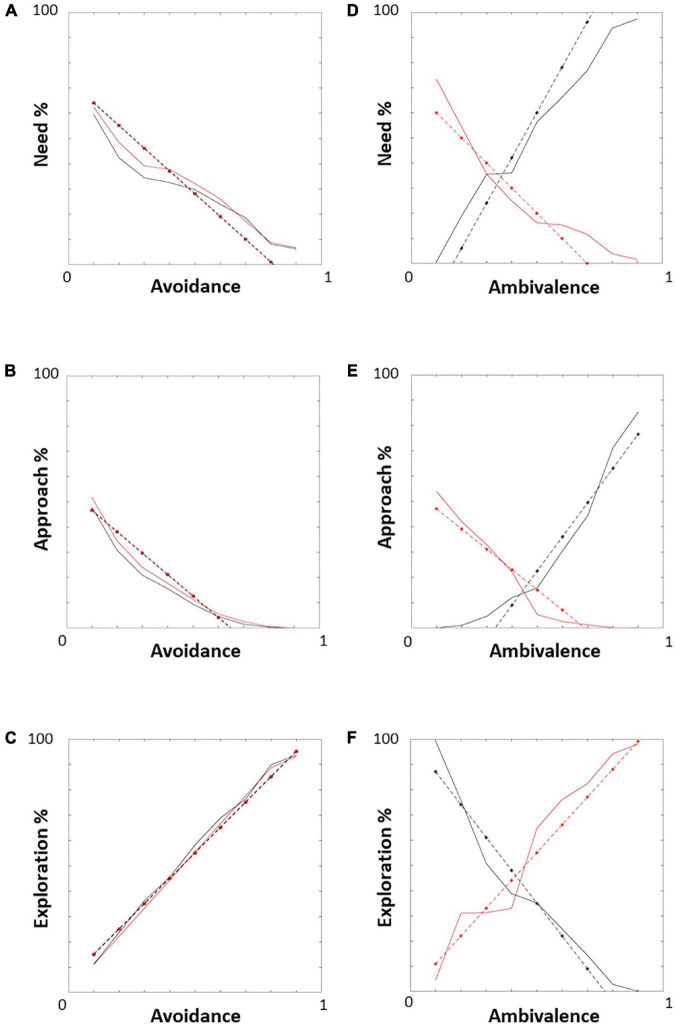
Need, approach, and exploration trends: simulated vs. expected. The graphs represent the simulated need, approach, and exploration trends (solid) compared to the expected ones (dashed; in black for the child, in red for the caregiver) in the avoidant **(A–C)** and ambivalent **(D–F)** cases. All simulated trends match those expected.

The compliance of the above results with what expected according to the literature (Figure 1) is confirmed by the comparison of simulated need, approach, and exploration trends (solid) with the expected ones (dashed; in black for the child, in red for the caregiver; Figure 13). Both in the avoidant (Figures 13A–C) and ambivalent (Figures 13D–F) cases, the simulated trends match those expected.

## Discussion

Attachment is a crucial and complex psychological phenomenon whose theory has been evolving for many decades, not only enormously widening its corpus but also refining its fundamental concepts and adopting different viewpoints (Fitton, 2012; Sutton, 2019). As a consequence, identifying a convenient conceptual basis on which to build a computational model of attachment has become increasingly difficult. We modeled attachment interactions computationally by relying on the most recent dimensional theory, thereby also testing it.

After reviewing some relevant previous models of attachment to identify their central features, we discuss below what we consider the contribution and limitations of our model, anticipating some future improvements.

### Previous models and their central features

Despite the psychological centrality of attachment, relatively few computational models have been created to study the phenomenon. They can be divided into (1) purely mathematical and (2) agent/robot-based models.

(1) Between those authors who adopt a purely mathematical stance, Buono et al. (2006) see child-mother interactions as a game in which the child behaves according to a payoff matrix. Following a categorical standpoint of attachment, they identify three possible kinds of game (i.e., attachment patterns): avoidant (in which the child does not approach), ambivalent (in which the child approaches and keeps guard), and secure (in which the child approaches). Also in accordance with the classical theory, Stevens and Zhang (2009) build a dynamic model considering attachment as the child’s system to regulate the distance from their secure-base caregiver. The authors assume that the child does that by using their physiological feedback and consider the child’s sensitivity in relation to the emission of opioids and norepinephrine. Thus, they identify three regions in the parameter space that represent each an attachment pattern: avoidance corresponds to high sensitivity to calming stimuli and low to arousing ones, while ambivalence corresponds to low sensitivity to calming stimuli and high to arousing ones. In the secure condition, sensitivity to both is low. Finally, Talevich (2017) sees attachment as a complex system that generates an attachment pattern as an emergent property. Three categories are identified, depending on the caregiver’s response: (1) from a response that becomes less and less frequent, the child learns to be avoidant, (2) from a constant response, the child learns to be secure, and (3) from an unpredictable response, the child learns to be ambivalent.

(2) As Petters and Waters (2015) discuss, ABMs have demonstrated to be a valuable choice to simulate attachment. Petters and Beaudoin (2017) describe an ABM underpinned by the CogAff (Sloman, 2008), an architecture developed to implement both cognitive and affective phenomena. Again, following the classical theory, attachment is seen as a system whose goal is maintaining an adequate distance from the secure-base caregiver. The child learns an optimal distance – defined by a Safe Range Limit (SRL) – during interactions, depending on the caregiver’s response to requests for care. An attachment pattern is determined so that: (1) when the caregiver’s responses come frequently on time, the SRL is large and the child secure, (2) when the caregiver’s responses come frequently late, the SRL is little and the child insecure. A step beyond the ABM is the robotic implementation, where the agents are enhanced with some kind of physical features. Likhachev and Arkin (2000) pioneered this field by making a robot explore an open environment with the constraint of feeling uncomfortable when beyond a given distance from an object. Amengual (2009) modeled then the attachment relationship through a 3D simulation tool, implementing an SSP-room populated by a robotic mother and child. In this case, the author simulates the classical secure pattern by endowing the mother with fixed behaviors and leaving the child free to attach and explore according to their perceived safety. Finally, experiments have also been made concerning affective bonds in human-robot interaction, which can be considered indispensable for integrating a robot in a human environment (Kaplan, 2001). Cañamero et al. (2006) implemented a perception-action architecture able to make a robot-child attach to a specific human-caregiver, thereby establishing the necessary connection for subsequent dimensional acquisition. Hiolle et al. (2012) have investigated a more complex scenario where the robot-child explores a play mat guided by the responses of their human-caregiver, implementing a secure-base dynamics.

Overall, these works have remarkably contributed to attachment modeling and provided a valuable basis for our model. However, our review allows for identifying two features that such models generally share and – we suggest – may have hindered their efficacy. (1) Most models refer to the early conceptualization of attachment that represents it as a categorical phenomenon (as opposed to dimensional). As a result, avoidance and ambivalence are considered aspects of the same dimension, which derive from the same caregiving feature. In contrast, as discussed above, the most recent research shows that they are independent dimensions, which should be modeled as corresponding to different caregiving features. Therefore, a model that considers avoidant and ambivalent patterns as generated by the same caregiving feature is expected to miss capturing some relevant characteristics of the relationship. (2) Most models focus on the behavioral (as opposed to representational) aspects of attachment. As a result, proximity is taken as the set-goal that drives action. In contrast, if, as discussed above, attachment has a dimensional (i.e., representational) nature, dimensions should be the control parameters that drive action. In this case, a model that focuses on behavior and does not explicitly consider the multiple dimensions involved is expected to suffer from related limitations. Therefore, the early categorical-behavioral approach seems incompatible with the dimensional-representational one. Since, as we demonstrate here, the DP is implementable and leads to simulations compliant with available attachment data, adopting an earlier theoretical perspective may limit modeling effectiveness.

### Contribution of our model

Starting from theoretical considerations, we implemented the Dimensional Attachment Model (DAM), an ABM that, following the most recent *dimensional perspective* (DP; (Fraley and Spieker, 2003; Gagliardi, 2021), separately reproduces the avoidant and ambivalent patterns generated by a child-caregiver dyad. Compared to the models that implement a categorical-behavioral perspective, the DAM differs by: (1) Considering independent attachment dimensions (avoidance and ambivalence) related to specific caregiving features (insensitivity and unresponsiveness, respectively); (2) Making the system work as a controller whose set-goals are the stored levels of such dimensions. The consistency of the simulations with what attachment literature allows us to expect supports the validity of the DP.

#### Psychological and behavioral variables

In the DAM, psychological variables – in the mind of the agents – and behavioral variables – observable in the lab – are distinguished. From the basic setting of two autonomous dot-like agents moving in a limited space, two measurable features that can be interpreted by the child as cues for the construction of dimensional levels (i.e., psychological representations) are selected:

1. In the avoidant case, the caregiver’s indifference (*i*) – the proportion of explorations of the caregiver (behavioral) – is considered. From this measure of the caregiver’s insensitive attitude, the child extracts a level of emotional separation (*S*_*E*_; psychological). The idea is that a mother’s decision to explore can be seen by the child as a sign of her active rejection – evolutionarily, a sign of her unwillingness to invest in her offspring (Chisholm, 1996; Chisholm and Sieff, 2014).
2. In the ambivalent case, the caregiver’s distancing (*d*) – the distance between the caregiver and the child (behavioral) – is considered. From this measure of the caregiver’s unresponsive attitude, the child extracts a level of perceived distance (*D*_*P*_; psychological). The idea is that a mother’s distance can be seen by the child as a sign of her impossibility to attend in case of need – evolutionarily, a sign of her inability to invest in her offspring (Chisholm, 1996; Chisholm and Sieff, 2014).

Therefore, from two behavioral variables, two corresponding psychological variables are derived (Equations 8, 9) – through a formula that is expected to depend on the agents and interaction context. The attacher uses these psychological variables (*S*_*E*_, *D*_*P*_) as dimensional levels to be compared with the corresponding stored dimensional set-goals (*T*_*E*_, *T*_*P*_) and select an action. Therefore, attachment works as a multidimensional control system (representations are compared to drive action).

#### Motivational dynamics

In our model, the agents are driven by intrinsic motivations – the child by attachment and exploration, the mother by caregiving and exploration. Moreover, each agent’s need is influenced by the other’s (coupled Equations 1–2 and 3–4), thereby creating an intertwined dynamics between the motivational systems. In this respect, a relevant role is played by the time spent without giving care – implemented by an iteration counter (*K*) – which is the main determinant of cycles of attachment and caregiving activations alternated by exploration. In fact, the interplay between attachment and exploration is central to the infant’s attachment patterns (Bowlby, 1969/1982; Ainsworth et al., 1978; Hesse, 2008).

#### Results

Simulations show that the DAM reproduces the quality expected by real avoidant and ambivalent relationships. Increasing the dimensional levels, children go from being “anti-avoidant” or ‘anti-ambivalent’ to secure to highly avoidant or ambivalent (Figures 8, 9). The DAM covers a broader range of cases compared to the standard theory (Ainsworth et al., 1978; Main and Solomon, 1990; Hesse, 2008), suggesting that extremely low dimensional levels (*A*_*v*_ = 0.1, *A*_*m*_ = 0.1) may correspond to rare instantiations of dysfunctional conditions – such as particular cases of compulsive dependence or self-reliance (Bowlby, 1973; Fonagy et al., 2002; Beck et al., 2015) – usually not considered for attachment classification. On the other hand, mid-levels (*A*_*v*_ = 0.5, *A*_*m*_ = 0.4) correspond to secure attachment, which is taken as the healthy standard, reflected in an optimal balance between attachment and exploration, where the child explores while being taken care of from time to time. Finally, the highest dimensional levels (*A*_*v*_ = 0.9, *A*_*m*_ = 0.9) strikingly represent the quality of the extreme avoidant and ambivalent relationships. The essence of these patterns is visually emphasized by the child’s and mother’s trajectories in the lab (Figure 10), which reflect the avoidant dyad’s independence and the ambivalent attacher’s over-involvement in the relationship related to their mother’s lack of care (Hesse, 2008; Mikulincer and Shaver, 2016; cf. attached video clips, see text footnote 9). The adherence of the DAM to attachment phenomena is further illustrated by the agents’ need as a function of the stored dimensional level (Figures 11A, 12A) and by the corresponding approach and exploration rates (Figures 11B, 12B). When the level raises, the attacher’s need for care decreases in the case of avoidance and increases in the case of ambivalence. At the same time, the avoidant explorations and the ambivalent approaches surge. Attacher’s and caregiver’s curves show matching trends, which entirely correspond to those expected (Figure 13).

The compliance of the DAM – in terms of need, approach, and exploration trends – with the expected attachment patterns demonstrates that such patterns can be generated by different dimensions. For each dimension, a specific configuration of agents’ goals needs to be considered. In particular, the high rate of child’s exploration in the avoidant case is the consequence of similar goals of high emotional separations. On the other hand, the high rate of child’s approaches in the ambivalent case is the consequence of opposite goals in terms of perceived distance. In other words, these outcomes can involve different areas of the relationship rather than be produced by opposite levels of the same dimension, as assumed by the early attachment theory.

Finally, it should be noted that, in the presented model, the drive-equations’ terms *c*_*0a,av*_, *c*_*0c,av*_, *c*_*0a,am*_, *c*_*0c,am*_ were kept constant for simplicity. However, the form of such equations suggests that those terms are to be expected to depend on the dimensional levels *A*_*v*_ and *A*_*m*_. Indeed, when *K* is zero (i.e., care is provided), the equations become:


$$a_{a\,v}\,\left[n+1\right]=C_{f,a\,v}\,\left(1-N_{G,a\,v}\right)\,S_{E\,a}\,\left[n\right]+c_{0\,a,a\,v}\,\left(1^{\prime }\right)$$

$$c_{a\,v}\,\left[n+1\right]=C_{f,a\,v}\,\left(1-N_{R,a\,v}\right)\,S_{E\,c}\,\left[n\right]+c_{0\,c,a\,v}\,\left(2^{\prime }\right)$$

$$a_{a\,m}\,\left[n+1\right]=C_{f,a\,m}\,N_{G,a\,m}\,D_{P\,a}\,\left[n\right]+c_{0\,a,a\,m}\,\left(3^{\prime }\right)$$

$$c_{a\,m}\,\left[n+1\right]=C_{f,a\,m}\,\left(1-N_{R,a\,m}\right)\,D_{P\,c}\,\left[n\right]+c_{0\,c,a\,m}\,\left(4^{\prime }\right)$$

and, all other things being equal (i.e., *N*, *S*_*E*_, *D*_*P*_), the drives will drop differently for different levels of avoidance and ambivalence. The drop will be greater for a more avoidant child (smaller *c*_*0a,av*_) and smaller for a more ambivalent child (greater *c*_*0a,am*_). Therefore, choosing appropriately variable coefficients is expected to further improve modeling performance. In fact, in the avoidant case, the following simple linear relationships:


$$c_{0\,a,a\,v}=-0.30\,A_{v}+0.60$$

$$c_{0\,c,a\,v}=-0.30\,A_{v}+0.59$$

**FIGURE 14:**
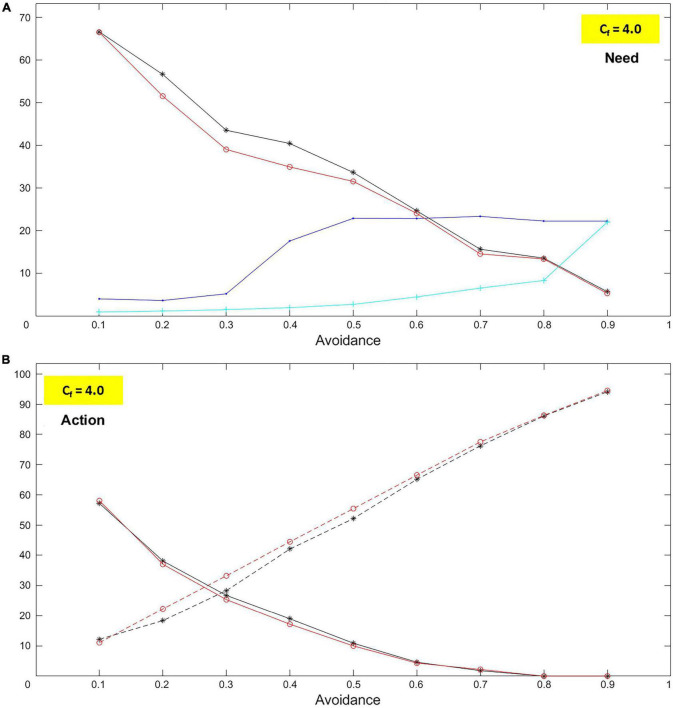
More accurate avoidant implementation. When variable *c*_0*a*,*av*_ and **c*_0*c*,*av*_* are considered, simulation performance improves in terms of **(A)** needs and **(B)** action (approaches and explorations).

– that implement the predicted kind of variability – enhance the system’s capacity to reproduce avoidance, as proven by the corresponding augmented range of needs, approaches, and explorations (Figure 14) compared to the above-illustrated case of constant *c*_*0a,av*_ and *c*_*0c,av*_ (Figure 11).

In conclusion, the presented DAM provides first computational support to the DP, suggesting it to be a convenient theoretical standpoint for attachment computational modeling. Adopting this perspective should help solve the limitations inherent to the early theory. This model also confirms the adequacy of the ABMs for the investigation of attachment (Petters and Waters, 2015).

### Limitations and future work

We want to stress that, since the DAM is the first computational model to aim at implementing the above-discussed DP, further studies in this direction are essential to confirm the presented results. Given this necessary premise, we can finally discuss six limitations of our work, which suggest future upgrades.

(1) The DP computational implementation was pursued with no other constraints than compliance with the theory. As a result, the DAM’s design is original, and the system expresses a non-linear dynamics that is not trivial to study. A programmed next step is to develop a simplified continuous model – relying on the discrete version presented here – to study its full dynamics through the tools of dynamical systems theory (Thelen and Smith, 1994; Coleman and Watson, 2000; Fraley and Brumbaugh, 2004; van Geert, 2019). Moreover, many parameters of the current version could be potentially investigated, and the performed sensitivity analysis (cf. Appendix) represents an example of such an investigation. This effort should be extended in future work.
(2) Despite the advantages in terms of simplicity, a relevant limitation of our DAM is being a 2D-ABM with dot-like agents. Attacher and caregiver have no physicality and, therefore, a very limited capability to express attachment, caregiving, and exploration behaviors – which can, in reality, assume numerous and sophisticated forms (Bowlby, 1969/1982; Ainsworth et al., 1978; Sroufe, 1995; Mikulincer and Shaver, 2016). An upgraded version of the model – where additional attachment, caregiving, and explorative behaviors are implemented – could significantly improve simulations. Such a model could be a 3D-ABM or a robotic implementation.
(3) The evaluation approach adopted for this model is merely qualitative. And, therefore, to be considered only preliminary. A goal for future work is being able to compare simulations to quantitative measures in real situations – such as frequency of attachment and exploration behaviors in a given SSP episode. While the presented model only implements ‘*approach*’ and ‘*explore*’ in a squared space, this goal will require considering specific behaviors and context features. For example, attachment can be expressed through an approach, a cry, or a look, only to mention evident instances (see point 2). Moreover, the same dyad can manifest their characteristic interaction pattern in significantly different ways, which are nonetheless recognizable as belonging to the same category – e.g., the patterns infant and parent produce in an SSP room or at home (Ainsworth et al., 1978). Therefore, although some quantitative matches could be found even with this essential model, more complex ones – representing a wider variety of behaviors in a given context – will be required to perform a quantitative evaluation.
(4) Despite the variability ensured by multiple random adjustments (in the direction taken, for example), this implementation remains quite deterministic. In this regard, we can suggest at least two ways to simulate the observed variance more closely. First, a probabilistic ‘*interest function*’ for each exploration target could be implemented – i.e., how an object becomes more or less appealing given the situation (the distance of the agent, for example). This feature would add some contextual uncertainty that the current model lacks. Second, random object disposition and starting agents’ positions could also be added to account for the unpredictability of the environment and initial conditions. Testing the effects of these factors on the simulation outcomes would be particularly interesting. For example, Petters and Waters (2015) suggested the initial configuration may be crucial for the attachment learning process, which seems to contradict the expected caregiver’s capacity to compensate for possible unpredictable factors.
(5) Attachment relationships are part of our life, which, of course, can involve any motivation. This DAM only considers exploration as a non-attachment and non-caregiving motivational system. A more detailed model of attachment should implement a higher number of situations and corresponding motivations. Interesting cases to model would be dysfunctional child-mother interactions with, for example, inversion of attachment (where child and mother invert their motivational systems) or dominant/submissive behaviors (where the child uses the ranking motivational system; Hennighausen and Lyons Ruth, 2005; Crittenden, 2008; Lyons-Ruth and Jacobvitz, 2008; Liotti, 2011).
(6) Finally, it is important to note that there is reason to hypothesize attachment dimensionality to be higher than three (Gagliardi, 2021), and multiple dimensions could theoretically be active simultaneously (or, more probably, in a rapid sequence). Therefore, the DAM should be extended to implement these cases^10^. Despite this implementation not aiming to combine avoidance and ambivalence, the model can relatively easily be tweaked to allow for experimenting with the coexistence of multiple dimensions. In fact, as discussed above, avoidance and ambivalence are incompatible – since they have opposing effects in terms of attachment activation (deactivation vs. hyper-activation).^11^ But, extending the number of dimensions, concurrent activations could be considered. In particular, the presented framework allows for implementing the dimension phobicity (Gagliardi, 2022; see text footnote 10), and its interaction with avoidance or ambivalence could be simulated. In this regard, it is worth noting that – to support such an interaction – an adequate activation mechanism should also be implemented.

## Conclusion

Attachment is as essential to our socio-psychological life as it is difficult to conceptualize and model. Following the latest theoretical developments, we considered here a *dimensional perspective* (DP) of attachment and suggested it to be a more convenient approach to model the phenomenon computationally than the classical categorical perspective. We supported our hypothesis by implementing the DAM – a DP-informed ABM of attachment. Our simulations of avoidance and ambivalence match the literature descriptions of the children who develop such patterns, thereby confirming the implementability and validity of the DP. According to this view, attachment is primarily a multi-dimensional control system.

## Data availability statement

The original contributions presented in the study are publicly available. This data can be found here: https://github.com/marc-gglrd/AC_Lab.

## Author contributions

The author confirms being the sole contributor of this work and has approved it for publication.

## Footnotes

1 Here, we only use the term *ambivalence*, but ambivalent children are also described as anxious and resistant (Ainsworth et al., 1978). The word *resistant* refers to the typical attitude of the child to resist the caregiver’s offers of care, while *ambivalent* describes the apparent relationship of “*love and hate”* (seeking care and resisting it) that the child manifests with their caregiver. Despite patterns being very often characterized by some anxiety, we do not term this dimension *anxious* to avoid suggesting an inherent anxiety disorder. Since the “*love and hate”* aspect is often striking, we prefer *ambivalence* over *resistance*.

2 This counter-intuitive relationship is meant to represent a distinctive feature of ambivalence: The resistant, overtly “angry” nature of the ambivalent child. As apparent in the typical SSP reunion episodes (Ainsworth et al., 1978), when the child meets the caregiver, they protest their absence (i.e., a higher attachment activation/need). Therefore, the need increases when it should decrease. It is like the ambivalent child keeps quiet while waiting (“*You are not here. Well, I don’t care.*”), growing background anger that they release when the caregiver shows up (“*Where were you? Argh*”).

3 The need function (Equation 5) depends on the drive in a monotonous way. More precisely, when the drive (*a* or *c*) grows, then the need also grows. Since the drive has only positive additive terms, and *K* is an iteration counter, when it drops to zero (because care has been provided), then the drive (and so the need) drops. For example, *K* can go from 5 to 0, abruptly eliminating one significant term from the drive.

4 Since the avoidant child and the insensitive caregiver are expected to show similar exploration rates, this equation has been used as a simplified form of $i[n]=\frac{100\;(N_{ex,c}\left[n\right]+\;N_{ex,a}\left[n\right])}{2n}$ which explicitly shows the influence of both agents on *i*. The two equations provide qualitatively identical results.

5 The coefficients in these two expressions must ensure that *T*_*i*_ can be interpreted as a percentage and *T*_*d*_ as a valid distance in the simulation environment. The values 1.1 and 0.24 meet these requirements.

6 The term “speed” indicates the maximum space an agent can cover from one iteration to the other and, therefore, sets a limit on the distance the agent can travel in one iteration.

7 The model was implemented in MATLAB (the code is available on https://github.com/marc-gglrd/AC_Lab). In this version (AC_Lab 1.0), a dimension is activated when the program is launched (i.e., no activation rule is coded). The settings reported here correspond to those in the code.

8 It is worth noting that the simulated trajectories cannot be directly compared to those observed in the SSP room. There, mother and child express caregiving and attachment through many different behaviors (e.g., a facial expression, a cry), not only by approaching. On the other hand, the model only uses a change of position to manifest caregiving and attachment (see the limitations discussed below). Nonetheless, the simulated trajectories can be considered a representation of the diverse behaviors expressed in the SSP “translated” into simple changes of position.

9 Also available at https://github.com/marc-gglrd/AC_Lab/tree/main/OUTPUT_PLOTS_stored.

10 The implementation of phobicity is included in the provided MATLAB version (AC_Lab 1.0) (https://github.com/marc-gglrd/AC_Lab).

11 Interestingly, some rapid alternations between high-avoidance and high-ambivalence appear plausible – for example, when an angry protest (high ambivalence) seems to be expressed by a cold, rational criticism (high avoidance).

## Appendix

*C*_*f*_ *sensitivity analysis.* The model’s main Equations (1–4) contain a coupling factor (*C*_*f*_) that connects each agent’s need to the other’s. Here, a sensitivity analysis for such a factor, both in the avoidant (Equations 1–2) and ambivalent (Equations 3–4) cases, is presented. The influence of the factor (*C*_*f*_ > 0) was evaluated against the case of no coupling (*C_f_* = 0) taking into account the trends of need and actions (approach and exploration rates) of the agents across the entire range of parameter levels (*A*_*v*_ = {0.1, 0.2,…, 0.9},*A*_*m*_ = {0.1, 0.2,…, 0.9}).^12^

(1) *Avoidant case*. The need and action trends were analyzed for *C*_*f*_ in the range [0.0, 8.0]. In this case, following the psychological literature, the system is considered to be compliant with the expected behavior when the need has a descending trend, which corresponds to decreasing approach and increasing exploration rates. According to this criterion, the system showed to work even when decoupled, but improved its performance for increasing values of *C*_*f*_, reaching its best around *C*_*f*_ = 4.0. At this point, the need showed a steeper trend, which corresponded to a more marked difference in terms of approaches and explorations between the extremes of the parameter range (*A*_*v*_ = 0.1 and *A*_*v*_ = 0.9). A further increase of *C*_*f*_ yielded a degradation of performance, which was completely lost for *C*_*f*_ = 8.0 (no monotonic trends of the curves with no approaches for *A*_*v*_≥0.5). Therefore, the analysis showed a qualitative performance improvement of the coupled system compared to the decoupled one.
(2) *Ambivalent case*. The need and action trends were analyzed for *C*_*f*_ in the range [0.0, 6.0]. In this case, following the psychological literature, the system is considered to be compliant with the expected behavior when: (1) For the child, the need has an ascending trend, which corresponds to increasing approach and decreasing exploration rates; and (2) For the caregiver, the need has a descending trend, which corresponds to decreasing approach and increasing exploration rates. According to this criterion, the system showed not to work properly when decoupled (no monotonic trends of the curves with no approaches for *A*_*m*_≤0.4), but improved its performance for increasing values of *C*_*f*_, reaching its best around *C*_*f*_ = 2.0. At this point, the need and action rates showed the expected trends. A further increase of *C*_*f*_ yielded a degradation of performance, which was completely lost for *C*_*f*_ = 6.0 (need practically constant, saturated at its maximum, for *A*_*m*_≥0.4). Therefore, this analysis also showed a qualitative performance improvement of the coupled system compared to the decoupled one.
